# STIM Proteins and Glutamate Receptors in Neurons: Role in Neuronal Physiology and Neurodegenerative Diseases

**DOI:** 10.3390/ijms20092289

**Published:** 2019-05-09

**Authors:** Karolina Serwach, Joanna Gruszczynska-Biegala

**Affiliations:** Molecular Biology Unit, Mossakowski Medical Research Centre, Polish Academy of Sciences, 5 Pawińskiego Str., 02-106 Warsaw, Poland; kserwach@imdik.pan.pl

**Keywords:** STIM, glutamate receptors, SOCE, synaptic plasticity, neurodegenerative diseases

## Abstract

Neuronal calcium (Ca^2+^) influx has long been ascribed mainly to voltage-gated Ca^2+^ channels and glutamate receptor channels. Recent research has shown that it is also complemented by stromal interaction molecule (STIM) protein-mediated store-operated Ca^2+^ entry (SOCE). SOCE is described as Ca^2+^ flow into cells in response to the depletion of endoplasmic reticulum Ca^2+^ stores. The present review summarizes recent studies that indicate a relationship between neuronal SOCE that is mediated by STIM1 and STIM2 proteins and glutamate receptors under both physiological and pathological conditions, such as neurodegenerative disorders. We present evidence that the dysregulation of neuronal SOCE and glutamate receptor activity are hallmarks of acute neurodegenerative diseases (e.g., traumatic brain injury and cerebral ischemia) and chronic neurodegenerative diseases (e.g., Alzheimer’s disease and Huntington’s disease). Emerging evidence indicates a role for STIM proteins and glutamate receptors in neuronal physiology and pathology, making them potential therapeutic targets.

## 1. Introduction

l-Glutamate is the most abundant neurotransmitter in the mammalian central nervous system (CNS) that mediates excitatory synaptic transmission [1]. It interacts with both ionotropic and metabotropic receptors, which belong to the group of glutamate receptors. Ionotropic receptors are ligand-gated ion channels that include α-amino-3-hydroxy-5-methyl-4-isoxazolepropionic acid receptors (AMPARs), *N*-methyl-d-aspartate receptors (NMDARs), and kainate receptors. Among all of the glutamate receptors, AMPARs, which consist of four subunits (GluA1–4), are considered the most significant mediators of excitatory neurotransmission in the CNS [2]. NMDARs, composed of three subunits (NR1–3), are involved in various processes, from learning and memory to neurodegeneration [3]. A single NMDAR is generally considered to consist of two glycine-binding NR1 subunits and two glutamate-binding NR2 subunits and is responsible for synaptic transmission and plasticity. Metabotropic glutamate receptors (mGluRs), which are classified into three groups (I, II, and III), have a widespread distribution in the CNS and play a pivotal role in synaptic transmission and activity-dependent synaptic plasticity. The main role in synaptic plasticity is assigned to group I mGluRs (mGluR1 and mGluR5) [4]. The binding of glutamate to group I mGluRs activates two main signaling pathways [5]. In the first pathway, group I mGluRs couple to Gα-protein, which activates phospholipase C (PLC), thus inducing the formation of inositol trisphosphate (IP_3_). IP_3_ interacts with the IP_3_ receptor (IP_3_R), causing the release of Ca^2+^ from endoplasmic reticulum (ER) stores [5]. The second pathway is associated with the formation of slow excitatory postsynaptic potentials [6] and is mediated by Gα-protein and transient receptor potential channel 3 (TRPC3) [7].

The stimulation of glutamate receptors plays a pivotal role in the formation of basal excitatory synaptic transmission and different forms of synaptic plasticity, such as long-term potentiation (LTP) and long-term depression (LTD). Both LTP and LTD are fundamental neuronal mechanisms that underlie learning and memory [8] and rely mainly on NMDAR activation. mGluRs have also been implicated in LTP and LTD, but the mechanism of this involvement is much more elusive [9,10]. NMDA-mediated LTP is described as enhancement of the synaptic response to baseline stimuli that occurs primarily as a result of cooperation between AMPARs and NMDARs. Synaptic activity initially stimulates the influx of sodium ions (Na^+^) through AMPAR channels. The flow of Na^+^ enhances the concentration of positively charged ions in the cytoplasm, causing cell depolarization. Consequently, NMDAR channels become permeable to calcium ions (Ca^2+^), and Ca^2+^ influx induces a cascade of plastic changes. In the initial stages of LTP (i.e., early LTP [E-LTP]), it activates Ca^2+^/calmodulin-dependent protein kinase II (CaMKII), protein kinase C (PKC), protein kinase A (PKA), and tyrosine kinase, which then phosphorylate AMPARs and NMDARs. The AMPA channel properties and activity-dependent synaptic delivery are regulated by phosphorylation at the Ser-831 and Ser-845 sites of the C-terminal cytoplasmic tail of GluA1 [11]. The first site is phosphorylated by CaMKII and PKC, which modulate the single-channel conductance of AMPARs [12]. The second site is phosphorylated by cyclic adenosine monophosphate (cAMP)-dependent PKA, which promotes the surface delivery of AMPARs [13] and increases the probability of receptor channel opening [14]. The transport of AMPARs to the synaptic site requires AMPARs-containing vesicles/endosomes and SNARE-proteins that mediate the fusion of vesicles/endosomes with the PM. Firstly, the receptors are inserted into the PM in the soma or dendrites at extrasynaptic sites and then they travel to dendritic spines [15]. NMDARs are phosphorylated by tyrosine kinase (which increases the open time of the channels) and PKC (which enhances the probability of channel opening and reduces their affinity to magnesium ions) [16]. Late LTP (L-LTP) is induced by changes in gene expression and by the synthesis of proteins that sustain LTP. These proteins contribute to increases in the number and surface area of dendritic spines and their postsynaptic sensitivity to neurotransmitters. The latter may also be associated with an increase in AMPAR synthesis [17].

Synaptic plasticity in the hippocampus, the neocortex, and other regions of the brain depends mainly on synaptic size and the content of AMPARs. Long-term potentiation at many central synapses depends on the delivery of AMPARs (i.e., exocytosis) that contain GluA1 to the postsynaptic site [18,19] and is linked with an increase in dendritic spine size [20]. Long-term depression is associated with the removal of AMPARs (i.e., endocytosis) from the synapse and shrinkage of dendritic spines [21]. Long-term depression may occur both presynaptically through the alleviation of glutamate release or postsynaptically through the internalization of AMPARs. There are two types of LTD: NMDA-dependent and mGluR-dependent [22]. NMDA-LTD is evoked by low-frequency stimulation. Slow Ca^2+^ flux through NMDARs activates calcineurin (CaN) phosphatase, which dephosphorylates the GluA1 subunit of AMPARs at Ser-845, causing the endocytosis of receptors [23]. mGluR-LTD is mediated mainly by group I mGluRs, but the mechanism is much more elusive [24]. The dysfunction of mGluR-LTD (but not NMDA-LTD) is associated with learning impairment in mouse models of aging and several neurodegenerative disorders [10]. Molecular entities that are implicated in synaptic plasticity-induced Ca^2+^ signaling include not only glutamate receptors but also depolarization-activated channels (e.g., voltage-gated Ca^2+^ channels [VGCCs]) and ER Ca^2+^ release via IP_3_Rs and ryanodine receptors (RyRs) [25].

Although glutamate plays an important role in physiological conditions, excessive glutamate concentrations that result in glutamate excitotoxicity can also cause the dysfunction and degeneration of neurons. Glutamate is the main excitatory neurotransmitter in the CNS, and its effects are far-reaching. Acute CNS insults, including traumatic brain injury (TBI) and cerebral ischemia, have been studied in this context. Glutamate excitotoxicity is also associated with chronic neurodegenerative disorders, including, among others, Alzheimer’s disease (AD), Parkinson’s disease (PD), and Huntington’s disease (HD) [11]. Prolonged exposure to glutamate and the associated excessive influx of ions into the cell can lead to Ca^2+^ overload [26,27]. In the CNS, Ca^2+^ influx is mediated mainly by VGCCs and ionotropic glutamate receptors, such as NMDARs and AMPARs [28]. It is also complemented by stromal interaction molecule (STIM)-dependent store-operated Ca^2+^ entry (SOCE) [28]. 

**Figure 1:**
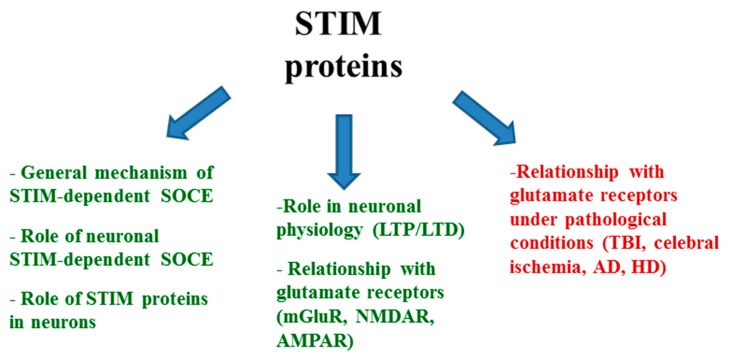
STIM proteins in neurons.

The present review provides an overview of the current state of knowledge on STIM proteins and SOCE contribution to the regulation of neuronal Ca^2+^ homeostasis under physiological conditions (e.g., synaptic plasticity, synaptic transmission, and trafficking) and under pathological conditions (e.g., TBI, cerebral ischemia, AD, and HD) in the context of the relationship between STIM proteins and glutamate receptors. A detailed plan of our study is presented in Figure 1.

## 2. STIM Proteins under Physiological Conditions

### 2.1. STIM Proteins and Intracellular Ca^2+^ Regulation

**Figure 2:**
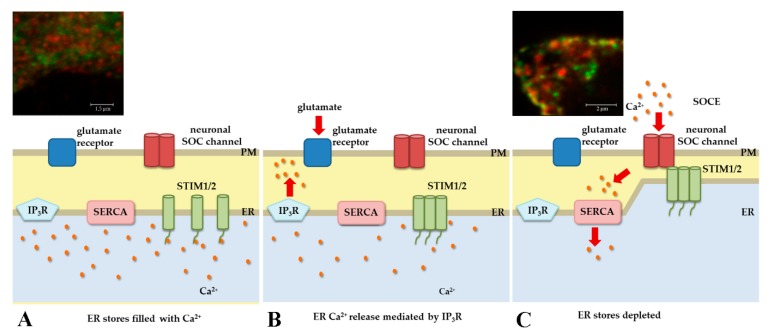
Model of coupling between STIM1/2 proteins and Orai channels during SOCE. (**A**) In the absence of external stimuli, STIM1/2 proteins are equally distributed throughout ER membrane and ER Ca^2+^ concentration is high. (**B**) After glutamate activation, IP_3_R depletes ER Ca^2+^ stores. Ca^2+^ dissociates from STIM1/2 N-terminal domain which results in STIM1/2 oligomerization and translocation to the PM. (**C**) STIM1/2 binds to Orai1, thereby activating SOCE. Then, SERCA pump transports Ca^2+^ from cytoplasm to ER to refill the stores with Ca^2+^. (**A**,**C**) The upper panels show confocal images of neurons co-expressing Orai1 (red) and YFP-STIM1 (green) before (**A**) and after store depletion by thapsigargin (**C**) where the proteins are redistributed equally and colocalized forming the complexes (yellow), respectively (modified from images in [41] with permission from Elsevier).

Two STIM isoforms, STIM1 and STIM2, are Ca^2+^ sensors that are localized in the ER, but STIM1 was also found in the plasma membrane (PM) [29]. In resting cells, with the ER full of Ca^2+^, STIMs have diffuse localization (Figure 2A). The release of Ca^2+^ from the ER into the cytoplasm results in STIM protein oligomerization (Figure 2B). Then the oligomers migrate to ER–PM junctions and interact with Orai1-3 Ca^2+^ channels (Figure 2C). Interactions between STIMs and Orais lead to the formation of large complexes that are visible under microscope as distinct puncta [30,31]. As a result of this interaction, Ca^2+^ flows from the extracellular space into the cytoplasm in a mechanism called SOCE (Figure 2C) [32,33]. The sarco-endoplasmic reticulum Ca^2+^-adenosine triphosphatase (SERCA) pump then transports Ca^2+^ to the ER, thus refilling ER stores [34]. 

SOCE is the main Ca^2+^ entry pathway into non-excitable cells [35,36,37]. Accumulating evidence also indicates its significant role in neurons in different regions of the CNS, including the hippocampal pyramidal neurons [38,39,40], cortical pyramidal neurons [41,42], and cerebellar Purkinje neurons [5]. STIM-mediated Ca^2+^ influx was shown to contribute to cellular and systemic phenotypes also in Drosophila neurons [43]. Although both STIM isoforms are found in the CNS, STIM1 is the predominant isoform in the cerebellum [5,41,44], and STIM2 is more abundant in the hippocampus [44,45] and cortex [29,44]. Previously, we reported formation of complexes of exogenous [41] (see also Figure 2, upper panels) and endogenous [46] STIM isoforms with Orai1 and different roles of both STIMs in neuronal SOCE [42]. The function of STIM1 protein in SOCE is undeniable, but less is known about STIM2. STIM2 has lower affinity for Ca^2+^ and migrates to the ER–PM junction in response to small changes in ER Ca^2+^ levels [42,46,47,48]. However, the coupling of STIM2 and Orai1 is weak and results in poor channel activation [29,49]. For these reasons, STIM2 is thought to stabilize basal Ca^2+^ levels [42,46,47]. Recent studies have identified a role for STIM2 in the activation of STIM1 and STIM1/Orai1 coupling when ER Ca^2+^ levels are not sufficiently low to activate the STIM1 response [49]. After the minimal depletion of ER stores, STIM2 recruits and causes the transition of STIM1 to its active conformation, which enables STIM1 coupling and the activation of Orai1. This suggests that STIM2 may increase the sensitivity of SOCE and maximize Orai1 function when the stimulus intensity is low [49]. Another study found a weak association between STIM1 and Orai1 in rat hippocampal neurons that lacked STIM2, suggesting that STIM2 may also facilitate STIM1 and Orai1 co-localization [50].

New evidence suggests that neuronal STIM-mediated SOCE preserves ER Ca^2+^ levels, participates in the regulation of spine morphogenesis, influences neuronal Ca^2+^ dynamics during synaptic excitation, and regulates gene expression [51]. Although the influence of STIM1 on spine architecture is unclear [51], STIM2 was shown to maintain hippocampal postsynaptic mushroom spines [29,52]. Low ER Ca^2+^ content activates STIM2-mediated SOCE, which supports a constant level of the Ca^2+^-CAMKII complex and stabilizes mushroom spines that participate in memory storage [29]. In turn, STIM1 was shown to play a role in neurogenesis [53]. SOCE is the major Ca^2+^ entry pathway, which regulates gene expression and proliferation of neural progenitor cells (NPCs). Both the suppression and deletion of STIM1 or Orai1 significantly alleviate the proliferation of embryonic and adult NPCs [53]. *STIM1* knockdown also decreases the proliferation and early differentiation of human NPCs [54]. In addition to Orai activation, STIM proteins may induce Ca^2+^ influx via TRPCs [5,55]. TRPC1 and Orai1 activation is mediated by different STIM1 domains. TRPC1 function depends on Orai1-mediated Ca^2+^ influx, which triggers the recruitment of TRPC1 into the PM where it is activated by STIM1. TRPC1 is thought to modify the initial Ca^2+^ signal that is caused by Orai1 activation [55]. Moreover, two research groups independently discovered a direct interaction between STIM1 protein and L-type VGCCs [56,57]. According to these studies, STIM1 suppresses the depolarization-mediated opening of L-type VGCCs. Interestingly, it is mediated by the same domain that activates neuronal store-operated channels (SOCs) [58]. The influence of STIM1 on VGCCs is also associated with an increase in channel internalization from the PM. STIM1 was also shown to control the structural plasticity of L-type VGCC-dependent dendritic spines. The NMDAR activation of L-type VGCCs was postulated to trigger Ca^2+^ release from the ER, which in turn causes STIM1 aggregation and inhibits L-type VGCCs, thus enhancing ER spine content and stabilizing mushroom spines [59]. In turn, STIM1 in complex with TRPC1 was shown to associate and inhibit L-type VGCCs as Ca_V_1.3, which is essential for the protection of dopaminergic neurons in the substantia nigra region [60]. Loss of dopaminergic neurons leads to PD, however, the mechanism of its development is not fully understood. Neuronal death and degeneration seen in PD as well as in AD and HD may be caused by, among other things, the inhibition of the ubiquitin–proteasome system (UPS) [61]. Importantly, UPS regulates STIMs distribution and SOCE function [61,62]. This suggests that Ca^2+^ shortage is an early event in neurodegeneration associated with UPS inhibition observed in these diseases. The above results deliver some better insight into the contribution of STIM proteins in neurodegeneration mechanisms.

### 2.2. STIM Proteins and Their Relationship with Glutamate Receptors

Increasingly more research is focusing on the influence of STIM proteins on glutamate receptors. Ng et al. showed that the activation of group I mGluRs stimulates STIM1 oligomerization and its transport to the PM [63]. This is consistent with a study by Hartmann’s group, who discovered that STIM1 protein is responsible for mGluR1-dependent synaptic transmission in cerebellar Purkinje neurons (PNs) and controls cerebellar motor behavior [5]. In mice with the PN-specific deletion of STIM1, mGluR1-dependent signaling was abolished. Interestingly, both IP_3_-dependent Ca^2+^ release from the ER and TRPC3-mediated slow excitatory postsynaptic currents were impaired. The disruption of these two pathways abolished cerebellar motor behavior [5]. Our study revealed that AMPARs in primary rat cortical neurons can interact with STIM proteins in a SOCE-dependent manner, thus demonstrating that STIM proteins can induce Ca^2+^ influx not only via Orai and TRPCs, but also through AMPARs [64]. AMPAR antagonists inhibit SOCE, and SOCE inhibitors decrease AMPA-induced Ca^2+^ influx. Additionally, the induction of SOCE by thapsigargin (TG) results in both direct and indirect AMPAR activation. We also found that both STIM1 and STIM2 proteins cooperate with GluA1 and GluA2 subunits of AMPARs. Although these interactions occur mainly in pyramidal neurons, they may also occur in non-pyramidal cells [64]. Garcia-Alvarez et al. showed that STIM2 protein can interact with AMPARs in a SOCE-independent manner [65]. STIM2 induces the cAMP/PKA-dependent surface delivery of GluA1 through exocytosis and endocytosis. The authors suggested that STIM2 couples PKA to AMPARs and promotes the phosphorylation of GluA1 at Ser-845. The phosphorylation of Ser-845 is widely known to regulate the activity-dependent trafficking and surface delivery of AMPARs. Surprisingly, STIM2 and the phosphorylation of GluA1 at Ser-831 are negatively correlated. In STIM2-silenced neurons, the phosphorylation of GluA1 is increased at Ser-831. Altogether, these findings indicate that STIM2 regulates the phosphorylation of GluA1 at both Ser-845 and Ser-831 [65].

Importantly, both serine residues, Ser-845 and Ser-831, play a pivotal role in LTP and LTD (i.e., forms of synaptic plasticity that are responsible for learning and memory) [65]. Yap et al. confirmed the role of STIM2 protein in LTP and LTD at hippocampal synapses [66]. The authors also found that the phosphorylation of GluA1 at Ser-845 is only one of the mechanisms by which STIM2 influences AMPAR delivery during LTP. In mice that lacked the GluA1 Ser-845 phosphorylation site, LTP was unchanged [67]. Other mechanisms also control the synaptic delivery of GluA1 during LTP [68]. Yap’s group speculated that STIM2 may likely participate in these mechanisms [66]. Mice with double *Stim1*/*Stim2* conditional knockout (cKO) exhibited an enhancement of LTP, which was associated with increases in the phosphorylation of GluA1, the transcriptional regulator CREB, and L-type VGCCs at PKA sites [69]. The ablation of *Stim* genes in the forebrain results in spatial memory impairments, similar to such impairments that are caused by NMDAR blockade [45]. This indicates an inverse correlation between spatial learning/memory and LTP. An increase in cAMP/PKA signaling impairs learning and memory functions [69]. Baba et al. suggested that SOCE may impact LTP by influencing NMDARs [39]. The activation of NMDARs in pyramidal neurons results in SOCE activation. NMDAR stimulation leads to the recruitment of IP_3_, which in turn interacts with IP_3_R, causing Ca^2+^ release from ER stores and the activation of SOCE. SOCE inhibitors decrease NMDA-dependent Ca^2+^ influx and synaptic plasticity in the hippocampus [39]. Nevertheless, Emptage et al. showed that the depletion of ER stores triggered SOCE in presynaptic neurons, thus influencing the frequency of spontaneous neurotransmitter release [38]. Therefore, the possibility that SOCE inhibitors prevent LTP by affecting presynaptic SOCE cannot be excluded. The PN-specific deletion of STIM1 reduced neuronal excitability and weakened intrinsic plasticity, but it did not affect LTP. These results suggest that STIM1 in PNs is essential for intrinsic plasticity but not for synaptic plasticity [70]. Other studies also reported a role for STIM1 and SOCs in the plasticity and maintenance of dendritic spines [71,72].

In contrast to LTP, LTD is mediated by the endocytosis of AMPARs from synaptic sites. A decrease in NMDA-mediated LTD was recently reported in *Stim2* cKO mice [66]. Although the precise mechanism by which STIM2 influences LTD is unknown, it is likely associated with phosphorylation of the GluA1 subunit of AMPARs at Ser-845 [66]. Mice with a serine-to-alanine mutation at this site exhibited LTD impairment that resembled *Stim2* cKO mice [66]. In primary cortical neurons, SOCE is required for mGluR-dependent LTD [24]. The inhibition of SOCE causes the impairment of (*S*)-3,5-dihydroxyphenylglycine (DHPG)-induced LTD. The SOCE inhibitor YM-58483 disrupts DHPG-induced LTD and the maintenance of DHPG-mediated cytosolic Ca^2+^ signals. These results suggest that SOCE is essential for the activation of downstream mGluR effectors. On the other hand, Majewski et al. found that mGluR signaling is impaired in STIM1-overexpressing mouse hippocampal neurons, with no changes in LTP or basal synaptic transmission [73]. In these neurons, both electrically and chemically induced LTD was decreased [73]. L- and T-type VGCCs were shown to be important for mGluR-mediated LTD [74,75], and STIM1 protein was shown to inhibit L-type VGCCs [56,57]. Therefore, one speculation is that mGluR-mediated LTD impairment in STIM1-overexpressing mice is linked inter alia with the STIM1-induced inhibition of L-type VGCCs [73].

## 3. Relationship between STIM Proteins, Glutamate, and Glutamate Receptors under Pathological Conditions

### 3.1. Traumatic Brain Injury

**Figure 3:**
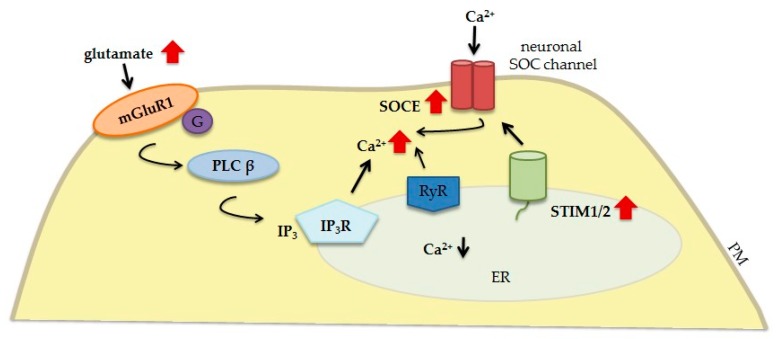
Contribution of neuronal STIM proteins and glutamate receptors to Ca^2+^ signaling dysregulation in TBI. The elevation of glutamate release leads to the excessive stimulation of glutamate receptors (e.g., mGluR1). mGluR1 couples to Gα-protein, which activates PLCβ and induces the formation of IP_3_ that interacts with IP_3_Rs, causing the release of Ca^2+^ from ER stores. mGluR1 also contributes to RyR-mediated Ca^2+^ leakage, resulting in lower ER Ca^2+^ levels, which in turn activate STIM proteins and promote SOCE that contributes to Ca^2+^ overload. Solid black arrows represent the interaction mechanisms; red solid arrows symbolize increased expression/concentration.

Traumatic brain injury (TBI) generally results from a jolt or blow to the head, but it may also be caused by a penetrating head injury [76]. It causes the disruption of normal brain function. Two kinds of damage can be caused by brain injury: primary damage and secondary damage. Primary damage occurs at the moment of injury. Secondary damage is initiated after the trauma and may last for several months or longer [77]. The most important pathological mechanism of secondary brain injury is an elevation of glutamate release, which can lead to the excessive stimulation of glutamate receptors (Figure 3). This is followed by an increase in cytoplasmic Ca^2+^ levels (i.e., Ca^2+^ overload), which in turn triggers downstream lethal cascades and is considered to be the main cause of neuronal apoptotic death after TBI. Ca^2+^ overload in TBI can be driven by both Ca^2+^ release from the ER and Ca^2+^ influx from the extracellular space through glutamate receptors, VGCCs or SOCE [78]. Interestingly, in an in vitro model of TBI, SOCE was enhanced 3 h after injury [79]. Numerous studies have demonstrated a significant contribution of mGluRs to glutamate-mediated pathological Ca^2+^ accumulation [3,80]. The glutamate-induced activation of mGluR1 is associated with both Ca^2+^ release from ER stores [11,81] and its influx through SOCs [82]. The former mechanism is mediated by IP_3_R- and/or RyR-dependent rapid Ca^2+^ release from intracellular stores. The latter mechanism operates as slow Ca^2+^ influx from the extracellular space. Since STIM1 is a Ca^2+^ sensor located in the ER that is known to play a role in neuronal injury, the influence of this protein on mGluRs has become an interesting focus of research. Hou et al. reported that mGluR-dependent IP_3_R- and/or RyR-mediated Ca^2+^ release from the ER after traumatic neuronal injury (TNI) in *STIM1*-deficient mice was significantly alleviated [82]. In the absence of STIM1, the ER is largely devoid of Ca^2+^; therefore, mGluR1-mediated signaling cannot induce Ca^2+^ release from intracellular stores. This mechanism indicates that STIM1 might link mGluR1 with its downstream effectors, which is consistent with previous findings [5]. The downregulation of reticulon protein 1-C (a member of membrane-bound proteins in the ER) was recently shown to protect cortical neurons against TNI by preserving intracellular Ca^2+^ homeostasis. This neuronal protection was associated with the inhibition of ER Ca^2+^ release via a mGluR1-dependent pathway and a reduction of STIM1 expression (with no effect on the expression of STIM2 or Orai1) and SOCE-mediated Ca^2+^ influx [83]. Therefore, STIM1 downregulation may inhibit apoptotic cell death after TBI and improve neuronal viability [82,83]. In contrast to the above findings, Rao et al. discovered that STIM2 expression was upregulated and STIM1 expression was unchanged after TBI in both in vitro and in vivo experiments [84]. The downregulation of STIM2 (but not STIM1) preserves neurological function and decreases apoptosis, thus improving neuronal survival. Neuroprotection that was conferred by decreasing ER Ca^2+^ release and reducing SOCE after STIM2 knockdown was associated with a reduction of Ca^2+^ overload. Since STIM2 is considered to support the development of secondary brain injury, the authors speculated that it may be a potential therapeutic target for the treatment of TBI [84].

### 3.2. Cerebral Ischemia

**Figure 4:**
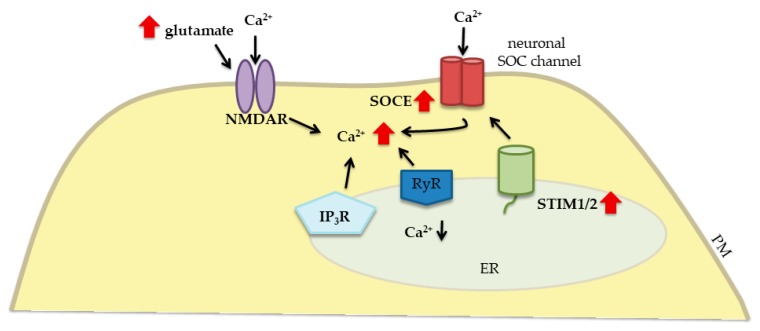
Contribution of neuronal STIM proteins and glutamate receptors to Ca^2+^ signaling dysregulation in cerebral ischemia. The elevation of glutamate release leads to the excessive stimulation of glutamate receptors (e.g., NMDARs). The overactivation of NMDARs results in excessive Ca^2+^ influx from both the extracellular space and ER stores, resulting in lower ER Ca^2+^ levels, which in turn promote STIM-mediated SOCE and contribute to Ca^2+^ overload. Solid black arrows represent the interaction mechanisms; red solid arrows symbolize increased expression/concentration.

Cerebral ischemia is a pathological condition that is caused by the insufficient supply of oxygen and nutrients to the brain, which may result from embolism, thrombosis, or systemic hypoperfusion [85]. Under low oxygen tension and low glucose levels, neurons lose their energy stores and become depolarized, while also releasing excessive glutamate into the extracellular space. The resulting overstimulation of glutamate receptors, particularly NMDARs, causes the excessive influx of Ca^2+^ ions (i.e., Ca^2+^ overload) and initiates ischemic brain damage and cell death [86] (Figure 4). Berna-Erro et al. found that STIM2 (but not STIM1) is essential for intracellular Ca^2+^ accumulation during cerebral ischemia [45]. Under hypoxic/hypoglycemic conditions, adenosine triphosphate-dependent Ca^2+^ transport to the ER is inhibited, which triggers persistent STIM2 activation and SOCE-mediated Ca^2+^ accumulation [29]. Importantly, SOCE may also trigger Ca^2+^ influx by increasing the release of glutamate and activating ionotropic receptors [45]. The combination of SOCE and glutamatergic Ca^2+^ inflow may rapidly increase Ca^2+^ concentrations to a dangerous level. Additionally, the lack of STIM2 decreases Ca^2+^ overload during an ischemic challenge. In acute hippocampal slices and hippocampal neurons in culture that were isolated from *Stim2* KO mice, SOCE is diminished and ER content is lower, so these animals are better able to survive hypoxic conditions. *Stim2* KO mice are better protected against cerebral ischemia compared with wildtype mice [45]. Another study suggested that STIM1 also contributes to pathological changes that occur in ischemia. The higher expression of STIM1 and Orai1 in the rat hippocampus after global cerebral ischemia was shown to enhance intracellular Ca^2+^ concentrations [87]. The resulting ischemia-induced neuronal death was attenuated by *Stim1* siRNA. The suppression of STIM1 in the early stage of ischemia attenuates neuronal death by inhibiting SOCE-induced neuronal apoptosis [87]. Moreover, mouse STIM1-deficient platelets were protected from neuronal damage after temporary cerebral ischemia [88]. These findings indicate that STIM proteins may be a new target for the treatment of ischemic stroke.

### 3.3. Alzheimer’s Disease

**Figure 5:**
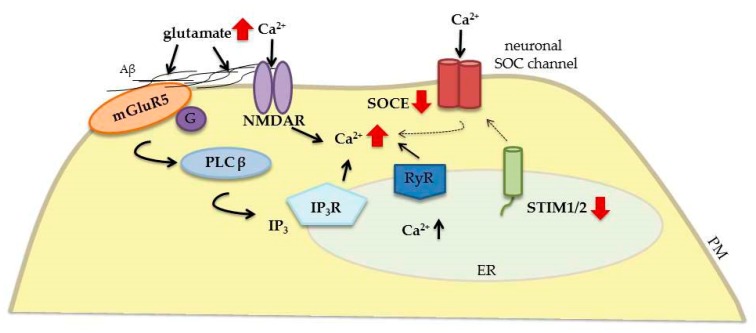
Contribution of neuronal STIM proteins and glutamate receptors to Ca^2+^ signaling dysregulation in AD. Elevations of both Aβ expression and glutamate release lead to the excessive stimulation of glutamate receptors (e.g., NMDARs and mGluR5). The overactivation of NMDARs results in excessive Ca^2+^ influx. In turn, mGluR5 couples to Gα-protein, which activates PLCβ and induces the formation of IP_3_, which interacts with IP_3_Rs and increases Ca^2+^ concentrations in the ER. Both pathways contribute to Ca^2+^ overload. The result of ER Ca^2+^ deprivation is STIM downregulation, which alleviates SOCE. Solid black arrows represent the interaction mechanisms, dotted black arrows mean attenuated mechanisms, and red solid arrows symbolize increased (up) or decreased (down) expression/concentration.

Alzheimer’s disease is the most common neurodegenerative disorder. Each year, it affects over 5 million people worldwide [28]. Alzheimer’s disease patients suffer from memory loss and cognitive impairment. In most cases, the first symptoms occur after 65 years of age (sporadic/late-onset AD). However, in some cases, the onset is earlier and is generally caused by mutations of genes that encode amyloid precursor protein (APP), presenilin-1 (PS1), and presenilin-2 (PS2; early-onset AD or familial AD [FAD]) [89]. Presenilins are enzymes that process integral membrane proteins, such as APP. Amyloidogenic APP processing results in the formation of neurotoxic forms of β-amyloid (Aβ). In AD patients, these peptides accumulate in extracellular plaques, causing neuronal death in the cerebral cortex and hippocampus. Nevertheless, recent research suggests that soluble Aβ oligomers, rather than amyloid plaques, cause neuronal dysfunction [28,90,91,92]. Busche et al. suggested that Aβ oligomers disrupt the balance between synaptic excitation and inhibition, resulting in the hyperactivation of cortical and hippocampal neurons and leading to Ca^2+^ overload [90] (Figure 5). β-amyloid enhances intracellular Ca^2+^ levels via multiple mechanisms. It affects synaptic NMDAR and mGluR5 activity [93,94,95], increases RyR-mediated Ca^2+^ leakage from the ER [96], and influences several Ca^2+^ entry pathways [97]. RyR-dependent Ca^2+^ release may be driven by Ca^2+^ influx via AMPARs, NMDARs, and VGCCs, especially in dendritic spines that lack IP_3_Rs [98]. Glutamate also activates mGluR1/5 and IP_3_/IP_3_R1-mediated Ca^2+^ release from the ER [99]. According to Zhang et al., hippocampal ER Ca^2+^ stores are refilled through the activation of mGluR5, which in turn leads to higher ER Ca^2+^ concentrations [94]. The refilling of mGluR1/5-mediated Ca^2+^ stores mainly depends on STIM1 [5]. In the cortex in sporadic AD patients, in the hippocampus in aged normal mice, and in a transgenic mouse model of FAD, ER Ca^2+^ overload results in the compensatory downregulation of STIM2 expression, impairments in synaptic SOCE, and lower CaMKII activity [52,94]. Insufficient CaMKII activation and an increase in CaN activity disturb the balance between LTP and LTD, facilitating LTD. The result is the destabilization of mushroom spines that are responsible for memory storage [99]. The pharmacological overexpression of STIM2 or inhibition of mGluR5 rescues synaptic SOCE and prevents the loss of mushroom spines in APP knock-in hippocampal neurons [94]. STIM2 protein also rescues CaMKII activity and protects dendritic spines against amyloid toxicity [94,100]. Presenilin-mediated synaptic deficits in AD were also postulated to be mediated by the dysregulation of neuronal SOCE [52,101]. In mouse embryonic fibroblasts with the lack of presenilins, STIM1 levels increased, whereas STIM2 expression decreased [102]. In these cells, SOCE was enhanced after Ca^2+^ store depletion. In turn, PS1 overexpression in human embryonic kidney 293 cells attenuated SOCE. Although no changes in STIM protein expression were observed in these cells, STIM2 expression decreased in human B lymphocytes with a PS1 mutation, which was paralleled by the alleviation of SOCE [102]. This is consistent with studies that were conducted with mouse hippocampal neurons. Mature spines from mutant PS1 mice exhibited a decrease in STIM2 expression and impairments in SOCE [52]. Impairments in SOCE and a reduction of synaptic STIM2 protein expression resulted in dendritic spine destabilization in mutant PS mice [52]. In mouse primary cortical neurons and human neuroblastoma SH-SY5Y cells, FAD PS1 increased γ-secretase cleavage of the STIM1 transmembrane domain, thus attenuating SOCE, which in turn destabilized dendritic spines. Both the overexpression of STIM1 protein and the inhibition of γ-secretase rescued dendritic spine loss [101]. Familial Alzheimer’s disease PS2, but not FAD PS1, in both SH-SY5Y cells and FAD patient-derived fibroblasts altered ER Ca^2+^ content by partially blocking SERCA activity [103]. Additionally, both FAD PS1 and PS2 mutants reduced SOCE, diminishing STIM1 expression [103]. In turn, Zhang et al. found that TRPC6 and Orai2 are neuronal SOCs that participate in SOCE in mature dendritic spines [104]. The knockdown of TRPC6/Orai2 resulted in SOCE inhibition and the loss of dendritic spines in wildtype neurons [104]. The TRPC6 activator hyperforin and novel neuronal SOC modulator NSN attenuated the loss of dendritic spines in APP and PS knock-in mice [104]. The stabilization of dendritic mushroom spines is considered to prevent memory loss in AD patients. Therefore, the modification of STIMs and SOCE may confer potential therapeutic benefit for the treatment of memory loss in AD patients.

### 3.4. Huntington’s Disease

**Figure 6:**
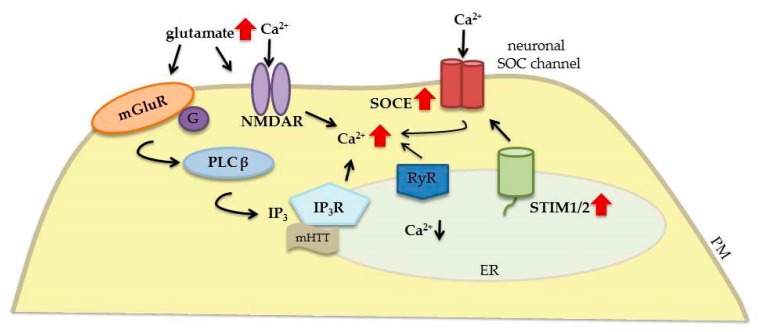
Contribution of neuronal STIM proteins and glutamate receptors to Ca^2+^ signaling dysregulation in HD. The elevation of glutamate release leads to the excessive stimulation of glutamate receptors (e.g., NMDARs and mGluRs). The overactivation of NMDARs results in excessive Ca^2+^ influx. In turn, mGluRs couple to Gα-protein, which activates PLCβ and induces the formation of IP_3_, which interacts with IP_3_Rs and causes the release of Ca^2+^ from ER stores. mHTT increases the sensitivity of IP_3_R1 to IP_3_, which additionally enhances ER Ca^2+^ release, resulting in a decrease in ER Ca^2+^ levels, which in turn activates STIM, promotes SOCE, and contributes to Ca^2+^ overload. Solid black arrows represent the interaction mechanisms; red solid arrows symbolize increased expression/concentration.

Huntington’s disease is an autosomal dominant neurodegenerative disorder that results from the expansion of a CAG repeat in the huntingtin gene (*HTT*), which is translated to polyglutamine (polyQ) in the huntingtin protein (HTT) [28,105]. The number of CAG repeats normally does not exceed 36; in HD patients, however, it is higher than 36. Mutant HTT (mHTT) causes the dysfunction of striatal neurons, synaptic loss, and eventually neuronal degeneration [106]. Neurodegeneration initially occurs mainly in the striatum and cortex. However, in later stages of the disease, it extends to various brain regions [107,108]. Huntington’s disease is characterized by numerous changes at the molecular level, including disturbances in Ca^2+^ homeostasis [105,109]. Studies of transgenic mice with mHTT revealed that NMDAR activation, accompanied by excitotoxicity and an increase in intracellular Ca^2+^ levels, was a significant pathogenic event in HD [105,110] (Figure 6). Elevations of Ca^2+^ concentrations also resulted from Ca^2+^ efflux via RyRs and IP_3_R1 [110] and the activation of group I mGluRs [105,111]. In striatal medium spiny neurons (MSNs) that were isolated from YAC128 HD transgenic mice, mHTT bound to IP_3_R1 after mGluR1 activation, increasing its sensitivity to IP_3_, which persistently reduced ER Ca^2+^ levels [106]. In MSNs, the decrease in ER Ca^2+^ concentration overactivated synaptic SOCE and enhanced STIM2 expression, which resulted in the disruption of dendritic spines [106,112]. In YAC128 MSNs, the knockdown of IP_3_Rs or STIM2 was shown to normalize SOCE and prevent dendritic spine loss, thus resulting in neuroprotective effects [106]. In addition to STIM2, the knockdown of STIM1, TRPC1/TRPC6, and Orai1/Orai2 rescued spine loss in YAC128 MSNs but did not affect the spine density of wildtype MSNs [112]. Recent studies showed that the expression of huntingtin-associated protein 1A (HAP1) was elevated in the striatum in a mouse model of HD [113]. HAP1, similar to mHTT, activated SOCE by influencing IP_3_R1 [113,114]. In human neuroblastoma cells, the HD pathological phenotype was mediated by expression of the N-terminal fragment of mHTT, which increased SOCE in a STIM1-dependent manner [115]. Similar results were observed in primary mouse cultures of MSNs and in mouse neuroblastoma cells, where the N-terminal HTT fragment enhanced SOCE through STIM1 and Orai1 or TRPC1 [116]. Moreover, tetrahydrocarbazoles were shown to attenuate the enhancement of SOCE in MSN cultures from transgenic YAC128 mice. The dysregulation of Ca^2+^ homeostasis is considered a pathological hallmark of HD, and these compounds may be leading molecular structures for the treatment of HD [110]. Additionally, the SOCE inhibitor EVP4593 rescued spine loss in both in vitro and in vivo HD models, normalizing neuronal SOCE and exerting neuroprotective activity against glutamate excitotoxicity [106,116,117]. The pharmacological modulation of SOCE and its components may have neuroprotective effects in HD patients.

## 4. Concluding Remarks

The experimental evidence that was reviewed in this paper clearly demonstrates a relationship between neuronal SOCE that is mediated by STIM proteins and glutamate receptors under both healthy and pathological conditions. Under physiological conditions, STIM1 mediates mGluR1-dependent synaptic transmission in cerebellar PNs, thus influencing cerebellar motor behavior [5]. In primary rat cortical neurons, SOCE is mediated by AMPAR channels that interact with STIM1 and STIM2 proteins [64]. The relationship between STIM proteins and glutamate receptors was also observed in different forms of synaptic plasticity, such as LTP and LTD (i.e., the mechanisms that are responsible for learning and memory) [65,66,69,72,73]. In primary rat hippocampal neurons, STIM2 induces the phosphorylation and surface delivery of the GluA1 subunit of AMPARs, which in turn contributes to LTP [65]. SOCE may also influence LTP by influencing NMDA receptors [39,59]. SOCE inhibitors decrease NMDA-dependent Ca^2+^ influx and synaptic plasticity in the hippocampus [39]. STIM proteins also contribute to both NMDA- and mGluR-mediated LTD, which is associated with the endocytosis of AMPARs from synaptic sites [24,66]. Although the precise influence of STIM proteins on LTD is unknown, it is probably associated with phosphorylation of the GluA1 subunit of AMPARs.

The relationship between neuronal SOCE and glutamate receptors is also observed in both acute neurodegenerative disorders (e.g., TBI and cerebral ischemia) and chronic neurodegenerative disorders (e.g., AD and HD). Nevertheless, changes in neuronal SOCE may be in opposite directions in different pathological conditions. SOCE appears to be neuroprotective in AD, whereas the blockade of this process may be neuroprotective in other neurological disorders, such as TBI, cerebral ischemia, and HD. In TBI, cerebral ischemia, and HD, neuronal ER Ca^2+^ stores are excessively depleted, and such depletion is associated with STIM overexpression and SOCE overactivation (Table 1). In TBI, it is mainly attributed to the stimulation of mGluRs. In cerebral ischemia, it is mainly attributed to the activation of NMDARs. In HD, it is attributed to both mGluRs and NMDARs [75,105,110,111]. The downregulation of STIMs expression eliminates the toxic effect of these proteins (Table 1). In contrast to TBI, ischemia, and HD, in AD neuronal ER Ca^2+^ stores are overfilled, which is assigned to mGluR and NMDAR activation [93,94,95]. STIMs and SOCE in AD are downregulated as a compensatory response to ER Ca^2+^ overfilling (Table 1). Although there are some reviews in literature that discuss the role of SOCE in neuronal physiology and pathology [28,34,92], this study focuses on the role of STIM1 and STIM2 proteins. Our paper not only summarizes and expands the function of SOCE and STIMs in neurons, but also indicates the relationship between STIM proteins and glutamate receptors in this case. 

In conclusion, the present study reveals that the restoration of physiological neuronal STIM-dependent SOCE and the normalization of STIM protein expression and glutamate receptor activity may confer potential beneficial effects for the treatment of both acute and chronic neurodegenerative diseases. Nevertheless, this approach has limitations (e.g., toxic effects on neuronal cells from nontarget regions). Undoubtedly, further studies are required in this field.

## Figures and Tables

**Table 1:** Summary of the functional significance of neuronal STIM proteins in neurodegenerative diseases.

Disease	Pathological Mechanism	STIM Expression	Role of STIM	Effect	References
TBI	Glutamate toxicity mediated by mGluR	STIM1 is overexpressed	- Increasing SOCE-mediated Ca^2+^ influx - Contributing to Ca^2+^ overload	toxic (blockade of STIM expression -> protective)	[82,83]
		STIM2 is overexpressed			[84]
Cerebral ischemia	Glutamate toxicity mediated by NMDAR	STIM1 is overexpressed	- Increasing SOCE-mediated Ca^2+^ influx - Contributing to Ca^2+^ overload	toxic (blockade of STIM expression -> protective)	[87]
		STIM2 is overexpressed			[45]
AD	Glutamate toxicity mediated by mGluR and NMDAR	STIM1 is downregulated	- Reducing SOCE-mediated Ca^2+^ influx - Stabilizes dendritic spines	neuroprotective	[101]
		STIM2 is downregulated			[52,94]
HD	Glutamate toxicity mediated by mGluR and NMDAR	STIM1 is overexpressed	- Increasing SOCE-mediated Ca^2+^ influx - Contributing to Ca^2+^ overload - Disrupting dendritic spines	toxic (blockade of STIM expression -> protective)	[112,116]
		STIM2 is overexpressed			[106,112]

## Abbreviations

AD	Alzheimer’s disease
AMPARs	α-amino-3-hydroxy-5-methyl-4-isoxazolepropionic acid receptors
APP	amyloid precursor protein
CaMKII	Ca^2+^/calmodulin-dependent protein kinase II
CNS	central nervous system
ER	endoplasmic reticulum
HD	Huntington’s disease
HTT	huntingtin protein
IP_3_	inositol trisphosphate
LTD	long-term depression
LTP	long-term potentiation
mGluRs	metabotropic glutamate receptors
MSNs	medium spiny neurons
NMDARs	N-methyl-D-aspartate receptors
PD	Parkinson’s disease
PNs	Purkinje neurons
PS1	presenilin 1
PS2	presenilin 2
RyRs	ryanodine receptors
SERCA	sarco-endoplasmic reticulum calcium-adenosine triphosphatase
SOCE	store-operated calcium entry
STIM	stromal interaction molecule
TBI	traumatic brain injury
TNI	traumatic neuronal injury
TRPC	transient receptor potential channel
VGCCs	voltage-gated calcium channels
